# Positive Psychology and SLA Revisited: Unearthing Positive Emotions in EFL Classrooms

**DOI:** 10.3389/fpsyg.2022.922413

**Published:** 2022-06-27

**Authors:** Elnaz Oladrostam, Abbas Ali Rezaee, Musa Nushi

**Affiliations:** ^1^University of Tehran, Tehran, Iran; ^2^Shahid Beheshti University, Tehran, Iran

**Keywords:** IPPLL, PERMA, positive psychology, microsystems, personal epistemology

## Abstract

Positive psychology focuses on the constructive effects of positive emotions on human behavior. Considering the recent plethoric research on positive emotions and SLA, this study pursues two important aims. The first aim is to find out whether there is any significant disparity between EFL teachers' and learners' views on an inventory of positive psychology in language learning (IPPLL) which was fostered by researchers. The second goal is to find out whether teachers' practices conform to their views on IPPLL and those expressed in the interview which was conducted with them. To this end, a 54-item questionnaire was developed and the revised questionnaire was administered to 385 learners. The results indicated that the teachers, in comparison with the learners, scored higher on all categories of the IPPLL. Moreover, unlike what they had expressed on the questionnaire and in the interview, the teachers did not capitalize on positive emotions as evinced in both teachers' practice and learners' experience with learning English. Implications of the findings for teacher education courses are presented.

## Introduction

Research has acknowledged that second language acquisition (SLA) is an emotionally driven process (Bown and White, 2010; Richards, 2020). Much of the scholarship on the contribution of affect, however, has been previously focused on negative emotions (MacIntyre, 2017, 2021). In a similar vein, SLA theories are mostly concerned with how linguistic information can be transferred to the learner (see Mitchell et al., 2019). This becomes obvious if we consider the major attempts at exploring the nature of second language (L2) learning, namely the input, output, and interaction hypotheses, which have gained wide support among L2 experts. All these hypotheses are almost exclusively concerned with information transfer. Since SLA theories form the backbone of most studies which are conducted in relation to language acquisition, this obsession with the cognitive side of learning can result in the lack of sufficient attention to emotions and the significant role they can play in learning. Positive psychology (PP), which has emerged as a major force since the turn of the 21st century, focuses on the constructive effects of positive emotions (e.g., gratitude, kindness, optimism, zest, and joy) on learners' wellbeing and optimal performance. Research within PP has shown that positively primed individuals exhibit fewer symptoms of depression and are more engaged and more likely to find creative solutions to problems by seeing the big picture that is normally hidden to negatively primed individuals (Fredrickson, 2003; Seligman et al., 2005).

However, during recent years, a number of studies (e.g., MacIntyre and Dewaele, 2014; Oxford, 2014; Li et al., 2018; Fang and Tang, 2021; Jin and Zhang, 2021) have investigated positive emotions in the context of language learning. In spite of abundant number of studies which have been directed at positive emotions, Oxford (2016) still concedes that a host of factors including motivation, perseverance, and character strengths need further attention and consideration. Additionally, when we consider teacher education courses, we can observe the fact that teacher educators are partially oblivious to positive emotions when preparing preservice teachers. Little attention has been paid to positive emotions and drawing teacher students' attention to positive emotions like joy, optimism, hope, love of learning, etc. that can have heaps of beneficial effects on learning a language Therefore, the purpose of the following study is two-fold. First, it aims to assess EFL teachers and learners' responses to the inventory of positive psychology in language learning or IPPLL to find out their views about the significance of positive emotions in learning and second, to investigate the teachers' practices in order to ascertain whether their practices and views about language teaching and learning confirm their responses to IPPLL. In other words, the researchers were trying to delve deeply in to the teaching moments to assess whether teachers pay adequate attention to positive emotions to find out whether they needed further instruction on embedding PP principles practically in their EFL classes.

## Review of The Literature

### Positive Psychology and PERMA

Positive psychology examines the influence of positive emotions like gratitude, optimism, zest, and joy on the efficient individuals' performance. Research has revealed that people who are more positive tend to be more innovative and have more creative solutions at their disposal in comparison to other people (Fredrickson, 2003; Seligman et al., 2005). Adapted theory of wellbeing has been put forward by Seligman (2011). According to this theory, happier people and those who experience wellbeing are more dynamic, creative, strategically equipped and therefore tend to have broadened states of mind. In other words, they tend to come up with more options in times of difficulty and conflict and therefore they will be more successful through the resources they create using the already existing options they possess. Seligman, a leading figure in PP, says the acronym PERMA (i.e., Positive Emotions, Engagement, Relationships, Meaning, and Accomplishment/Achievement) and the list of character strengths have been used to define characteristics of happy and successful people. In other words, those people who exhibit PERMA are happier and therefore more successful people. In a similar vein, many studies (e.g., MacIntyre and Vincze, 2017; Dewaele and Alfawzan, 2018; Khajavy et al., 2018, 2021; Ebn-Abbasi et al., 2022) found positive correlations between positive emotions, motivational constructs and willingness to communicate (WTC) which are considered as crucial factors in language learning.

Concerning the beneficial effects of positive emotions in terms of more creativity, broadened states of mind, and success defined as better test results or motivational constructs that are likely to affect language learning indirectly, positive emotions need to be further investigated to get a deeper understanding of their impact on language learning. In recent years, many studies have investigated positive emotions in relation to SLA, but some positive emotions have not yet been fully explored and need to be examined thoroughly in relation to linguistic performance and achievement. This can be certified through Oxford (2016) theoretical framework named EMPHATICS which comprises nine dimensions. Those dimensions are emotion and empathy, meaning and motivation, perseverance, agency and autonomy, time, hardiness, intelligences, character strengths, and self-factors including self-efficacy, self-concept, self-esteem, and self-verification. She believes that many EMPHATICS themes such as meaning, empathy, hope, optimism, hardiness, habits of mind, and character strengths have not been discussed in relation to SLA. She goes further to state that other themes like resilience and intelligences have rarely been addressed in the field.

### Positive Psychology and SLA

Although positive emotions have received scant attention previously, they have been considered and scrutinized very closely in SLA recently. For instance, MacIntyre and Vincze (2017) conducted a study on the relationship between the ratio of positive and negative emotions and motivational constructs that drive learners to acquire a language. The results indicated that there were stronger correlations between positive emotions and motivational constructs such as integrative orientation, acculturation, L2 ideal self, and L2 ought to- self in comparison to negative emotions. That study showed the rather important role of positive emotions in cultivation of motivation which in turn lead to better learning of L2. MacIntyre (2021) affirms that studies examining PP interventions that can indirectly affect factors like willingness to communicate in the L2 (L2 WTC) are sparse. There are however researchers like Dewaele et al. (2019) who point out that some PP interventions have been carried out in educational contexts like universities and schools, with the results revealing that the interventions strengthened teachers' and learners' experiences of flow, hope, courage, wellbeing, creativity, happiness, resilience, and grit. Similarly, Mercer et al. (2018) reported that there is a growing body of research of PP in SLA. Dewaele and Alfawzan's (2018) study was an example of those research studies in which the researchers looked in to the effect of enjoyment and anxiety on language learning. The results of their study indicated that foreign language enjoyment (FLE) positively correlated with test results, whereas foreign language classroom anxiety (FLCA) negatively correlated with test scores. It was also shown that FLE tended to be more significantly related with proficiency in comparison with FLCA. In the qualitative phase of the study, it was also noted that the teacher's character not only made the class enjoyable, but it also led to better learning and better test results in learners' views.

Dewaele et al. (2019) maintains that around the turn of the millennium researchers developed a burgeoning interest in the role of emotions in both foreign language learning and teaching beyond the already established constructs like anxiety, motivation, and attitudes. As a result, a deep understanding of the role of positive as well as negative learners' and teachers' emotions has emerged. This understanding has led to more and more empirical research using a range of epistemological as well as methodological approaches. However, they distinguish between two periods, namely 2012 which witnessed the publication of a number of early studies in some peripheral journals and 2016 which was the actual starting point for the recognition of PP within the field of SLA followed by an ascending number of publications which related positive emotions to SLA in mainstream journals. Given the fact that focus of the copious number of studies within SLA field has been on the role of positive and negative emotions in learning and the fact that both teachers' and learners' emotions have been acknowledged and understood by researchers during recent years (Dewaele et al., 2019), this study probes the role of positive emotions in language learning from the vantage point of learners and teachers. Furthermore, considering the thin scientific knowledge base which informs teacher education courses (Darling-Hammond, 2016) and lack of the more useful innovative methods to be practiced by teachers and teacher educators (Barak, 2017), the current study examined EFL teachers' classes to observe coverage of positive emotions in their classroom.

### Recurrent Problems With Teacher Education Courses

According to McKeown (2014), “teacher education refers to both initial preparation of pre-service teachers and continuing professional development for in-service teachers” (p.128). She listed two reasons for why teacher education is of significance. First, teacher education courses professionalize the teaching field and affect students' learning. Second, they contribute to the overall success of schools. Teacher educators are considered as key players in this process. They provide professional development for in-service teachers, they constantly work to prepare novice teachers to enter the field and they consult schools when problems arise, they usually work within a national framework, they help in preparing curriculum, they revise assessment instruments, and they write textbooks.

McKeown (2014) certified that because changes across a system are very complex and convoluted and they necessitate a whole system change of assessment tools or curricular changes, nobody focuses on the whole system changes. For example, when curricular content changes, adaptations also need to be made to assessment tools and procedures. Nobody can teach in one way but test students in another way. Due to the complexities and intricacies involved in teaching a whole system, teacher educators only focus on one component which is teachers. McKeown (2014) mentioned that it is irrefutably true that providing teachers with short-term professional development in the hope that the whole system will change is erroneous. If changes across the system are warranted, professional development must be provided for headmasters, policymakers, financial officers, custodial staff and everybody who has a responsibility within that system.

Gage (1964) has attested that practice of teacher education courses was much stronger than even that of medicine ones. There are however two inherent problems with teacher education courses. First, there is a dearth of research about efficacious teaching that can form part of the teacher education courses. The second problem is the absence of research concerning different approaches to teachers' recruitment and preparation, a shortcoming which prevents a proper understanding of how teachers are educated. Similarly, he highlighted the very thin scientific knowledge base of teacher education courses. He noticed that most of the teacher training courses were guided by common sense rather than a good scientific base. To illustrate his point, he used some of the common sense findings. For example, it was concluded that in order to understand how much students have learned, teachers should subtract pre-test scores from post-test scores. Another example is the fact that in order for a particular behavior to develop it should be rewarded and in order to extinguish a particular behavior it should be punished. He concluded that all these common sense findings were the opposite of what had been found in education research.

Darling-Hammond (2016) agrees with the above-mentioned points. She contends that lack of respect for scientific knowledge in teaching and teacher education is real. She adds that although significant progress has been made toward achieving a sound scientific basis for teacher education courses, new knowledge has not been employed effectively by most of the teacher educators and policy makers. Similarly, Motoca et al. (2014) argue that a gap exists between research and practice. This gap highlights two interrelated issues. First, there are several obstacles on the way of development of interventions, their examination, and application in real world. Second, context of research and practice are different. Whereas context of research controls only ecological factors, context of practice should control all environmental factors and real world conditions which form classroom activities and students. Yet another problem with teacher education courses is teacher educators' unwillingness to encourage teachers to make real changes in their practice by exploiting the new technology or new models. A good example of a study on this issue is the one by Barak (2017) in which it was concluded that teacher educators did not provide effective and sufficient models for the strengthening of the reform-based practice.

### The Present Study

This study pursued two aims. First, it assessed EFL teachers and learners' responses to the inventory of positive psychology in language learning or IPPLL to find out their views about the significance of positive emotions in learning; second, it investigated the teachers' practices in order to ascertain whether their classroom practices and views about language teaching and learning would confirm their responses to IPPLL. In other words, the researchers were trying to delve deeply in to the teaching moments to assess whether the teachers paid adequate attention to positive emotions to establish whether they needed further instruction on embedding PP principles practically in their EFL classes. The study was conducted to answer these two questions:

1. Are there any significant differences in the way EFL teachers and learners approach positive psychology in relation to language learning?
2. Do teachers' classroom practices conform with their responses to the inventory of positive psychology in language learning (IPPLL) and interview questions?

## Method

### Participants

The study used a mixed-methods design. In the quantitative phase of the study, two groups of EFL learners filled in the Virtues in Action (VIA) questionnaire in the pilot and the main studies. The participants in the pilot study were 369, 120 males (32.5%) and 249 females (67.5%), with the average age of 18 years. They were studying English at a private English language institute in Tehran, Iran and their language proficiency ranged from pre-intermediate to upper-intermediate. Their levels of proficiency were determined through achievement tests they had taken in previous semesters and through their teachers' assessments of their language knowledge. They all spoke Persian as their native tongue. In the main phase of study, 385 learners, 70 males (17.6%) and 315 females (82.4%), with the same average age (*M* = 18) and proficiency levels participated. A group of 100 female teachers also participated in the main study. They were all EFL teachers at the institute. Their age ranged from 20 to 50. Two female teachers (named teacher A and teacher B in this research) also participated in the qualitative stage of the study. Teacher A was 45 years old and had 18 years of experience in teaching English as a foreign language (EFL). Teacher B was 48 and had 20 years of EFL teaching experience. Table 1 summarizes the participants' information. As has been pointed out 369 male and female students participated in the pilot phase of the study. EFL practitioners did not participate at this phase of the study due to their busy work related schedules. After administering the original version of the questionnaire and removing nine items, the revised questionnaire was again given to 385 male and female participants and 100 English practitioners. Moreover, two female teachers were asked to take part in the qualitative phase of the study. These two teachers were selected because they were judged to be the most veteran instructors at the institute and this allowed the researchers to witness a more comprehensive picture of what an efficient form of instruction would be like at the institute. The researchers had to exclude male teachers since in order to gain access to main branches of the institute in Iran, one needs to obtain legal permission and this permission was not granted to the researchers.

**Table 1:** Number of the participants in each phase of the study.

**Phases**		**Male**	**Female**	**Total**
Learners				
Quantitative	Pilot	120	249	369
	Main	70	315	385
Teachers				
Quantitative			100	100
Qualitative			2	2

### Instruments

The VIA inventory was first devised by Peterson and Seligman (2004). This inventory consists of six categories. The first category is *wisdom and knowledge* which includes items like creativity, curiosity, open-mindedness, love of learning, perspective, and innovation. The second category is *courage* comprising items like bravery, persistence, integrity, vitality, and zest. The third category is *humanity* which includes items such as love, kindness, and social intelligence. The fourth category is *justice* encompassing citizenship, fairness, and leadership. The fifth category is *temperance* which covers forgiveness and mercy, humility, prudence, and self-control. Finally, *transcendence* category comprises appreciation of beauty and excellence, gratitude, hope, humor, and spirituality. The selected version of the questionnaire which was used in this study was the adapted version by Ruch et al. (2014). In comparison to the previous version which was devised in 2004, Ruch et al.'s version is more straightforward and simpler and includes more items which are related to language acquisition. For instance, integrity and vitality which are not closely related to SLA have been removed from the adapted version and an item like team work which has a closer symbiosis to language learning has been added. Furthermore, spirituality was replaced with a simpler and straightforward item, namely religiousness. *Wisdom and knowledge* in this questionnaire encompasses creativity, curiosity, open-mindedness, love of learning, and perspective. Compared with the VIA inventory, innovation is missing from the adapted version. *Courage* consists of bravery, perseverance, honesty, and zest with integrity and vitality being absent in the adapted version. *Humanity* encompasses love, kindness, and social intelligence, the same items included in the VIA version. *Justice* covers teamwork, fairness, and leadership, with the citizenship being replaced by team work in the adapted version. *Temperance* includes forgiveness, modesty, prudence, and self-regulation. The same items are present in the VIA inventory but with a slight change. For instance, instead of self-control and modesty, items like self-regulation and humility are seen. Finally, *transcendence* involves appreciation of beauty and excellence, gratitude, hope, humor, and religiousness. These items replicate those in the VIA inventory, with the exception of spirituality being superseded by religiousness. The researchers extracted all the character strengths from Ruch et al. (2014) inventory and related all PP strengths to language learning. For instance, creativity is explained as teachers' and learners' use of creative methods or love of learning is defined as students' interest in classmates, teacher, materials, or classroom atmosphere to relate it to language learning and the context in which it happens. After the development of the items, the newly designed questionnaire was given to a panel of 10 experts in positive psychology. They were all seasoned at writing related articles of positive psychology; they were also experienced English instructors and therefore had a deep understanding of the categories of positive psychology along with their component strengths. Moreover, the experts were cognizant of the significance of positive emotions in L2 learning. They were asked to judge if the items in each category of the IPPLL actually measured the strengths which were listed as subparts of each category in Ruch et al.'s (2014) inventory. After the experts judged the questionnaire items and confirmed the overall content and construct of the new instrument, the questionnaire was administered to the participants in the study. Further proof for the validity of the IPPLL was established through statistical measures which were conducted on the instrument, namely exploratory and confirmatory factor analyses.

The first 10 items of the questionnaire belonged to the first category or wisdom and knowledge. Items 1 and 2 were directed at creativity, items 3, 4, 5, and 6 measured love of learning, item 7 was related to curiosity, item 8 was related to open-mindedness, and items 9 and 10 measured perspective.

Items 11–18 formed the second category, namely courage. Items 11 and 12 were aimed at measuring bravery, items 13 and 14 measured perseverance and honesty respectively, and items 15–18 were directed at zest, the last constituent factor of the courage category.

Items 19–29 belonged to the third category or humanity category. Items 19, 20, 25, 26, and 27 were connected to social intelligence, items 23 and 24 measured kindness, and items 21, 22, 28, and 29 were directed at measuring love.

Items 30–34 belonged to justice category. Items 30, 31, and 34 investigated teamwork, items 32 and 33 were directed at fairness and leadership respectively.

Items 35–45 belonged to temperance category. Items 41, 42, 43, 44, and 45 measured self-regulation, items 35 and 39 measured prudence, and item 40 evaluated the effect of forgiveness on learning.

Finally, number 46 to number 54 belonged to the last category (i.e., transcendence category). Items 46, 47, and 48 were aimed at measuring gratitude, items 53 and 54 measured appreciation of beauty, items 50, 51, and 52 were directed at humor, and item 49 assessed the effect of hope (see Appendix C).

The researchers also conducted a structured interview with two teachers. The interview consisted of nine researcher-made questions which aimed at evaluating teachers' views about language learners, teachers and learning. The questions were designed in a way to extract the elements of PERMA and PP from the teachers' talks (see Appendix A). The researchers also used an observation checklist to observe the two teachers' classes. The observation checklist items were originally taken from the institute's observation lists, but the items were later adapted and modified to enable the researchers to observe possible flowering of positive emotions through teachers' classroom practices. For instance, an item like classroom arrangement was viewed and analyzed with respect to the space it provided for students to have more interactions with each other and to exchange more emotions. In a similar vein, use of games or group work and pair work were assessed using positive emotions criteria. For example, did the teachers create innovative and interesting games to make students more elevated, positive, and cooperative or did the teachers used pair work or group work that encouraged students to get closer to each other and to express their emotions more openly? Did such activities enhance students' emotional dispositions? Similarly, other items in the developed checklist were also examined meticulously. Did the practitioners create a positive learning environment or did they establish a positive rapport with learners? Did they use materials and realia like interesting pictures to create a general sense of enjoyment in learning? Did they use any specific instructional interventions to boost students MI (multiple intelligences) especially their emotional intelligence (EQ)? Did they use assessment or corrections efficiently so as to diminish students' stress and to increase students' sense of positivity? Did they decrease teachers' talking time (TTT) and increase students' talking time (STT) to augment students' sense of enjoyment and to decrease their anxiety and stress? Finally, considering items like homework or progress chart, did the teachers' evaluated students' progress both in terms of their learning and engagement and interest in English classroom or did the practitioners involve students in writing homework in the form of emotional portfolios as to empower them with tools to monitor and check students' emotional stance more closely in learning or did they only ask students to do conventional types of homework? (see Appendix B).

### Procedure

This study was carried out in two phases: the pilot study and the main one. The participants in the first phase were 369 Iranian EFL learners (mean age = 18) studying either at pre-intermediate or intermediate levels. In the main study, 385 EFL learners with the same mean age and proficiency levels participated. Before administering the questionnaire, the participants were informed that their identity would remain anonymous and the information they provided would not be shared with any third party. They were encouraged to read all the items scrupulously and to tick one of the boxes which applied to them. The questionnaire was first allotted to 369 learners. Then, an exploratory factor analysis (EFA) was conducted on the data from 350 learners since 19 participants either missed a majority of the items or gave irrelevant answers. The results showed that 9 items did not have factor loadings (3, 7, 14, 22, 25, 27, 38, 40, and 53). The results of the EFA also demonstrated that the six factors extracted by the principal axis factoring accounted for 52.08 percent of the variance. The confirmatory factor analysis established the construct validity of the IPPLL. Moreover, non-significant chi-square, ratio of chi-square over the degree of freedom, the root mean square of error approximation (RMSEA), and Holeter index of sampling adequacy all indicated that the model enjoyed a good fit. The revised questionnaire (see Appendix C) was distributed among 385 EFL learners in the main phase of the study, the answers provided by 361 participants were valid for analysis (data by 24 learners had to be excluded because of incomplete answers).

This revised questionnaire was then handed out to 100 teachers. Like what happened in case of administering the questionnaire to the learners, the teachers were told that their identities would not be disclosed and the information they provided would remain confidential. The participants were encouraged to go through all the items fastidiously and to tick one of the boxes which applied to them. Their answers were then compared with students' answers to the same items through relevant statistical analyses.

In the qualitative phase of the study, two teachers' classes were observed for two consecutive semesters which equal 6 months. The classes were observed twice during the 6 months to probe in to the teachers' teaching and learning practices conscientiously. The observations were unannounced to allow the researchers to observe the teachers' real practices in the classroom and see whether they heeded positive emotions during their teaching moments and whether PERMA can be extracted from their practice. After the observations, the researchers conducted an interview with the teachers. Moreover, the learners in the two teachers' classes were asked to write as much as they deemed necessary about their experiences with language learning, especially about their feelings about their teacher, how well they were learning English, their classroom activities, and their relationship with their classmates and their teacher.

## Results

### The Pilot Study

The first draft of the questionnaire was administered to 369 EFL learners out of whom 19 learners were excluded because they either missed the majority of the items or gave irrelevant answers (e.g., they had checked a similar choice across all the items of the questionnaire). The Cronbach's alpha reliability indices and exploratory factor analysis were run on the results obtained from the remaining 350 participants.

### Cronbach's Alpha Reliability Indices

Tables II–VII in Appendix D display the Cronbach's alpha reliability indices and item-total correlations for the six sub-categories of the IPPLL questionnaire. The Cronbach's alpha for the wisdom and knowledge category was 0.839. All of the items showed item-total correlations higher than 0.50, (i.e., large effect size) except for the third and the seventh items whose contributions to the total score were very low. Their correlations with the total score were 0.208 and 0.179 respectively (Table II). As shown in Table III, the second sub-category of the IPPLL questionnaire, courage, enjoyed a Cronbach's alpha reliability of 0.837. Item 14 had the lowest item-total correlation of 0.135 out of the eight items of this section of the questionnaire. The third sub-category of the IPPLL, humanity, had 11 items with a reliability index of 0.834. All of the items, except for items 22, 25, and 27, showed high (>0.50) item-total correlations whose contributions to the total IPPLL were 0.208, 0.184, and 0.183 respectively (Table IV). The fourth sub-category of the IPPLL questionnaire, justice, enjoyed a Cronbach's alpha reliability of 0.831. All of the items had large item-total correlations (Table V). The fifth sub-category of the IPPLL, temperance, had 11 items with a reliability index of 0.875. All of the items showed high (>0.50) item-total correlations; except for items 38 and 40 whose contributions to the total IPPLL were 0.159 and 0.123 (Table VI). Finally, as shown in Table VII, the transcendence was measured through nine items with a reliability index of 0.840. Item 53 was the only one with low contribution (i.e., 0.123 to the total score). Based on the results obtained from the pilot study, nine items including items 3, 7, 14, 22, 25, 27, 38, 40, and 53 did not have factor loadings and had to be removed from the original questionnaire. The revised questionnaire was then given to 385 participants.

### The Main Study

#### Exploratory Factor Analysis

**Figure 1:**
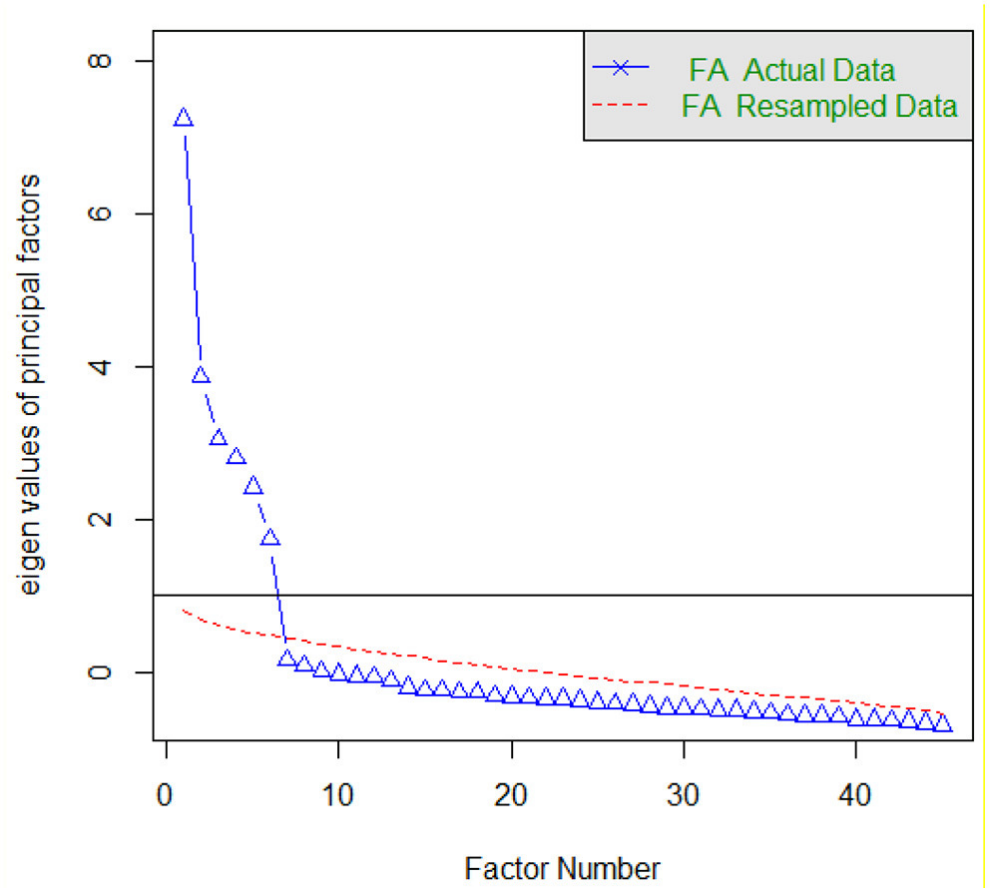
Parallel Analysis: Number of factors to be extracted for IPPLL questionnaire (main study).

An exploratory factor analysis using principal axis factoring (PAF) method was run to probe the underlying constructs of the 45 items of the IPPLL questionnaire. The Parallel Analysis in Figure 1 and the total variance explained in Table 2, suggested a six-factor solution which accounted for 52.08 percent of the total variance.

**Table 2:** Total variance explained (main study).

**Factor**	**Initial eigenvalues**			**Extraction sums of squared loadings**		
	**Total**	**% of Variance**	**Cumulative %**	**Total**	**% of Variance**	**Cumulative %**
1	8.042	17.870	17.870	7.568	16.817	16.817
2	4.520	10.045	27.915	4.071	9.047	25.865
3	3.972	8.827	36.742	3.497	7.771	33.635
4	3.734	8.297	45.040	3.242	7.205	40.840
5	3.354	7.454	52.493	2.849	6.331	47.171
6	2.687	5.971	58.465	2.211	4.913	52.084

#### Confirmatory Factor Analysis

**Figure 2:**
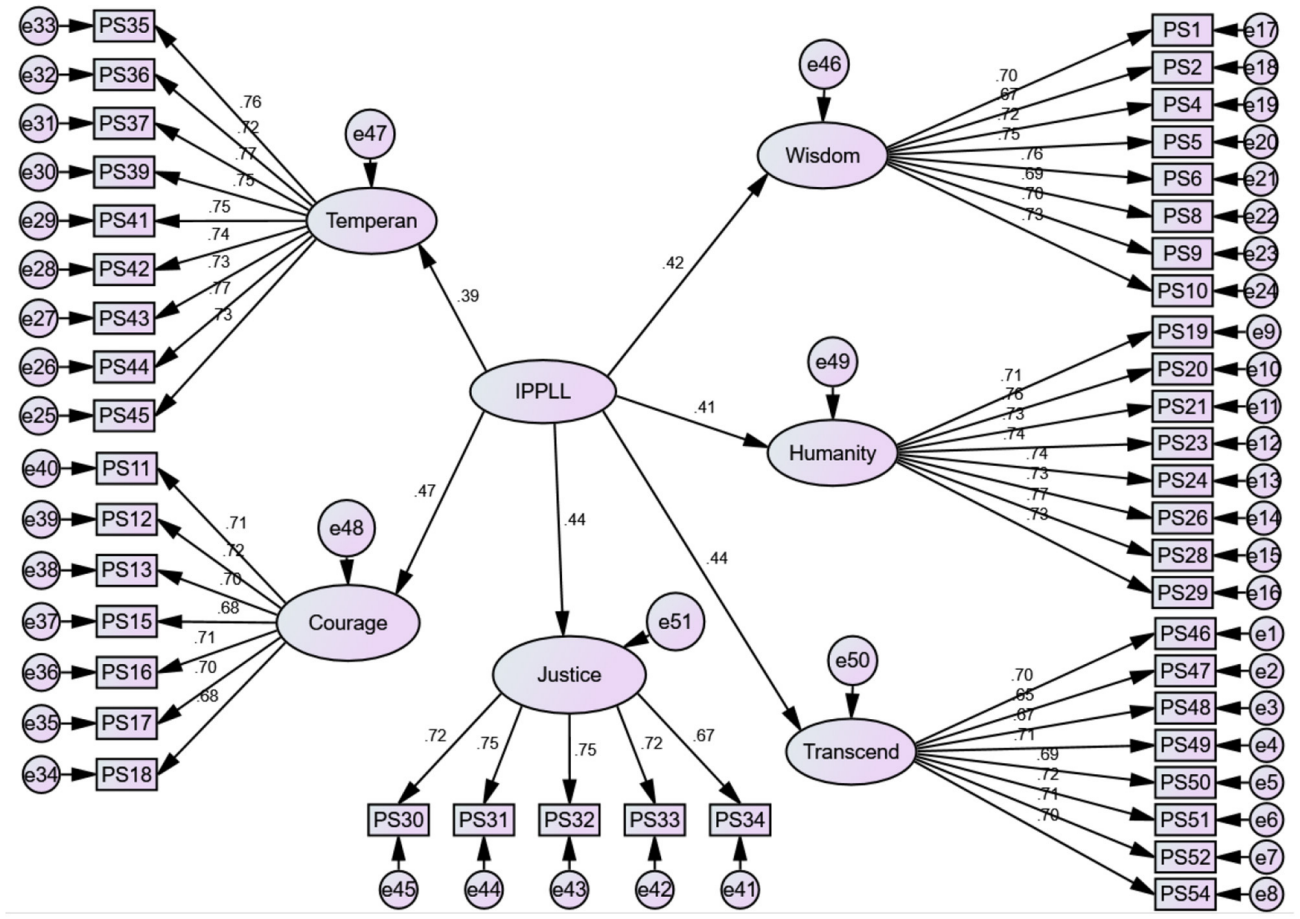
Trait structures of components of IPPLL questionnaire.

Figure 2 shows the CFA model probed in this study. The square rectangles represent the 45 items of the IPPLL questionnaire. Each sub-set of items measure a latent (unobserved) variable. The latent variables are represented by ovals. The single-headed arrows indicate direct relationship between the variables. The small circles connected to items are the error terms. The coefficients written on the single-headed arrows are analogous to standardized regression beta values. The results indicated that all of the 45 items of the IPPLL questionnaire had significant (Beta ≧ 0.30) contributions to their latent variables.

### Teachers' and Students' Views on IPPLL Construct

#### Normality of Data

In order to answer the first research question, a multivariate ANOVA (MANOVA) was run on the data (teachers' and students' responses to the revised questionnaire). Table 3 displays the skewness and kurtosis statistics for the teachers and students on six components of IPPLL. Since the absolute values of skewness and kurtosis statistics were lower than ±2 (Bae and Bachman, 2010), it can be concluded that the assumption of normality was retained.

**Table 3:** Testing normality of data.

**Group**		***N***	**Skewness**	**Kurtosis**
Students	Wisdom	361	0.186	−0.784
	Courage	361	0.032	−0.770
	Humanity	361	0.119	−0.662
	Justice	361	0.119	−0.875
	Temperance	361	0.138	−0.737
	Transcendence	361	0.076	−0.626
Teachers	Wisdom	100	−0.254	−0.605
	Courage	100	−0.378	0.787
	Humanity	100	−0.463	−0.039
	Justice	100	0.213	−0.836
	Temperance	100	−0.368	−0.452
	Transcendence	100	−0.034	0.265

#### Homogeneity of Variances

MANOVA assumes that the groups being compared do not have significant differences in their variances. The assumption of homogeneity of variances is tested through the Levene's statistic. Table 4 shows that the probabilities associated with the Levene's F-values were all higher than 0.05; hence homogeneity of variances of groups.

**Table 4:** Levene's test of equality of error variances.

	***F***	**df1**	**df2**	**Sig**.
Wisdom	1.869	1	459	0.172
Courage	3.220	1	459	0.073
Humanity	1.805	1	459	0.180
Justice	3.652	1	459	0.057
Temperance	1.986	1	459	0.159
Transcendence	2.703	1	459	0.101

#### Homogeneity of Covariance Matrices

MANOVA also assumes that the correlation between any two dependent variables is roughly the same across the group (i.e., homogeneity of covariance matrices). This assumption is tested through the Box' M. Table 5 indicates that the results of the Box' Test (*M* = 11.80, *p* = 0.951 > 0.001) was not significant. That is to say, the assumption of homogeneity of covariance matrices was met. It needs to be noted that the Box's M should be tested at 0.001 level (Field, 2013).

**Table 5:** Box's test of equality of covariance matrices.

Box's M	11.808
*F*	0.549
df1	21
df2	123732.647
Sig.	0.951

Table 6 displays the means of the teachers and learners' responses on six components of IPPLL. The results show that the teachers had higher means on all six sub-sets of IPPLL than the students. The results of MANOVA [*F*_(6, 454)_ = 20.04, *p* = 0.000, Partial η^2^ = 0.209 representing a large effect size] revealed that there were significant differences between the teachers and students' means on six components of IPPLL (Table 7).

**Table 6:** Descriptive statistics; components of IPLL by groups.

**Dependent variable**	**Group**	**Mean**	**Std. error**	**95% confidence interval**	
				**Lower bound**	**Upper bound**
Wisdom	Student	21.983	0.354	21.288	22.679
Wisdom	Teacher	26.600	0.673	25.278	27.922
Courage	Student	19.407	0.309	18.800	20.014
Courage	Teacher	23.510	0.587	22.357	24.663
Humanity	Student	22.022	0.361	21.312	22.732
Humanity	Teacher	26.640	0.687	25.291	27.989
Justice	Student	13.767	0.230	13.314	14.220
Justice	Teacher	16.610	0.438	15.749	17.471
Temperance	Student	24.684	0.407	23.884	25.485
Temperance	Teacher	30.030	0.774	28.509	31.551
Transcendence	Student	22.186	0.342	21.513	22.858
Transcendence	Teacher	26.860	0.650	25.583	28.137

**Table 7:** Tests of between-subjects effects; components of IPPLL by groups.

**Source**	**Dependent variable**	**Type III sum of squares**	**df**	**Mean square**	***F***	**Sig**.	**Partial eta squared**
Group	Wisdom	1668.993	1	1668.993	36.891	0.000	0.074
	Courage	1318.155	1	1318.155	38.274	0.000	0.077
	Humanity	1669.875	1	1669.875	35.428	0.000	0.072
	Justice	632.797	1	632.797	32.998	0.000	0.067
	Temperance	2237.845	1	2237.845	37.367	0.000	0.075
	Transcendence	1711.035	1	1711.035	40.498	0.000	0.081

**Figure 3:**
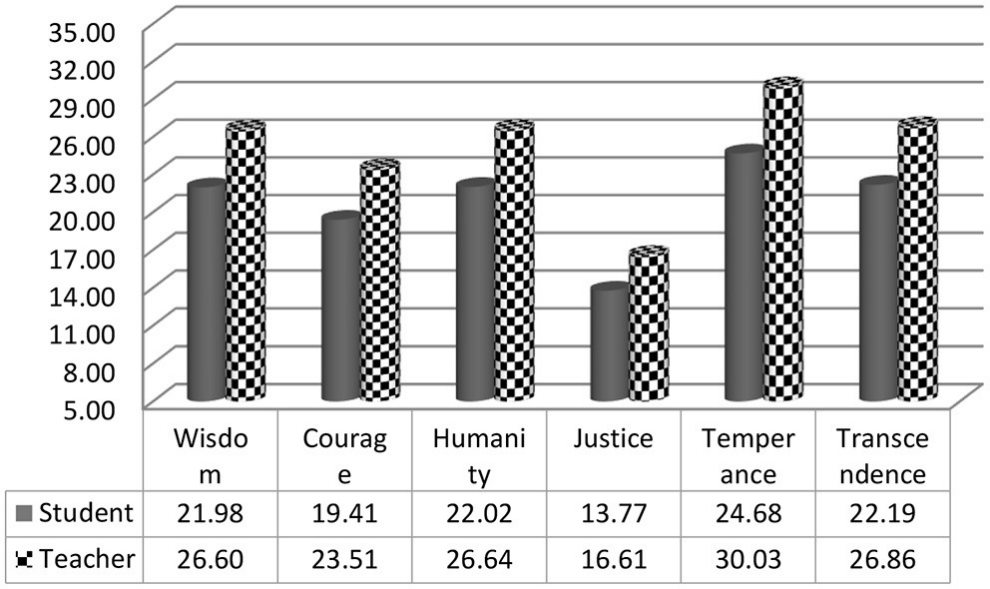
Means on components of IPPLL by groups.

As shown in Figure 3, the teachers had a higher mean in all items of IPPLL in comparison to the students.

### Conformity of Teachers' Views With Their Instructional Practice

In order to answer the second research question, the researchers used the data gathered from the two teachers' classroom observations and their interviews. Observation of teacher A's classes showed that she mostly tried her best to make sure that the learners had met the main objectives. For example, if the main objective was using simple forms of greetings, she attempted to provide the learners with ample practice and feedback to ensure the learners had a clear picture of various forms of greeting in their mind. However, the class was mostly teacher-centered. The teacher did not make an appropriate balance between her TTT and STT. The learners were mostly bored in her classes. It seemed that the teacher could not establish a positive rapport with the learners.

After conducting the interview with the teacher, many positive emotions such as creativity, love, zest, love of learning, and perseverance were found. It is necessary to point out that the criterion for finding positive emotions was the VIA inventory. Grounded theory was used to extract positive emotions and elements of PERMA from the teachers' talks. Grounded theory (Corbin and Strauss, 2008) is an approach in which the “explanatory categories emanate from the data rather than being pre-planned” (Oxford, 2014, p. 599). Therefore, the purpose here was to analyze teachers' responses and to extract the elements of PERMA and different positive emotions from teachers' talks. Teacher A's answers to the interview questions are as follows:

In your idea what makes students learn better?

*There are a lot of things that make students learn better. One of them is the teacher's* ***innovation**. The other factor is the* ***energy*** *that the teacher brings to the class. Yet* ***love*** *of teaching is also vital*.

What makes a teacher successful?

*When you* ***love*** *your job, you will be successful. When your students* ***see that you are*** ***trying really hard*** *to teach them, you are successful*.

What characteristics do all unsuccessful students share?

*They are* ***not attracted to learning**. They* ***don't love learning**. They are* ***bored*** *most of the time and they don't participate in classroom discussions. They are lazy and they don't study. They are not motivated*.

What characteristics do all successful teachers share?

*Their students* ***are attracted*** *to them. Their students* ***love*** *them. There is a great* ***relationship*** *between them and their students. Their students are really interested in the class and learning and come to class with full* ***energy!***

Some elements of PERMA were also found from the teacher's talks. Engagement was the first factor which was found as the first element.

What factors are deemed as important factors in teaching process?

***Engagement*** *of the teacher or how you involve students in the lesson matters. When the teacher goes to the class with* ***love*** *and* ***energy**. When she prepares everything in advance and when she thinks of* ***enjoyable*** *activities to be done in the class*.

As evident in the transcripts above, positive emotions like love, perseverance, and zest were taken out from teacher A's talks. Love is subcategorized under P part of the PERMA while perseverance and zest belong to the E part of PERMA. Love of learning, perseverance, creativity, and zest were also pulled out which are separate components of A part of the PERMA. Analysis of teacher A's answers to the interview questions as well as her teaching practices paved the way for the extraction of the elements of positive emotions and some parts of the PERMA including relationship, love, perseverance and zest but some important positive emotions like perspective which refers to the ability of providing others with appropriate forms of feedback, and fostering a sense of hope and gratitude were absent from the teacher's practices and her responses to interview questions. She even listed some negative emotions like students' weariness and apathy or lack of interest in learning which can have detrimental effects in terms of barricading learners' language acquisition journey.

Teacher B led a teacher-fronted class. When her classes were observed, the researchers discerned that the teacher was not successful in striking a balance between her TTT and STT. The researchers also wrote comments like ‘the teacher needs to add some flavor to her lessons' or ‘the classes are very monotonous' on the observation form. During her teaching moments, she only paid attention to cognitive factors and largely disregarded establishing a positive affinity with learners. However, during the interview, the teacher cited some positive emotions like love of learning, perseverance, and zest. Teacher B's answers to the interview questions are as follows:

What makes students successful learners?

*Students'* ***motivation*** *makes them successful. When they are motivated, they try to achieve their goals as best as they can*.

What factors make teachers successful?

*There are a lot of factors. A teacher needs to be* ***trustworthy**. The teacher should* ***respect*** *students. She should be close to learners and should call them by their first name. When you respect your students, a good communication and* ***relationship*** *is built between you and your students. That is the way through which learning takes place*.

What factors make teachers unsuccessful?

*Their students do not trust them. Their students feel distant. They therefore feel* ***anxious*** *and* ***stressed*** *most of the time and they don't have class participation*.

What characteristics do all successful students have in common?

*They are* ***highly motivated**. They are* ***hard-working**. They study so much and ask so many questions to make sure they have learnt everything correctly. They* ***love learning*** *and try to learn with pleasure*.

What factors are deemed as important factors in teaching and learning process?

*Being organized is the most important factor since it causes your students to be able to* ***trust*** *you. You should be punctual. You should teach them in an orderly way. Students will* ***enjoy*** *learning much better when they feel that they are moving step by step*.

What factors are usually ignored in teaching and learning process?

*Students* ***do not do their homework**. They make an alibi for not doing their homework. Anyway, they are students. Teachers most of the times forget that students need to have some* ***fun*** *in the classroom. They like games for example. Therefore, we should try to have some innovative forms of practice*.

Teacher B's answers exhibit most of the elements of PERMA and positive emotions. For example, the teacher elaborated on students' having fun or students' merriment in the class when they either played games or when teaching was done in a stepwise fashion. The teacher even elucidated the impact of negative emotions like anxiety or stress which caused the learners to eschew speaking in the class. Comparisons between the learners' and teachers' views to the items of IPPLL also suggested that teachers had a very favorable view toward positive emotions but when their classes were observed, they failed in attending to positive emotions which could play a facilitative role in students' learning experience.

Unlike their responses to IPPLL items, practitioners who took part in this study took no heed of positive emotions during their teaching moments. There were no special use of games or pair work or group work with the aim of establishing a better affinity among learners. If pair work or group work were included, the only purpose was doing trivial learning tasks such as doing exercises of the book with no opportunities for genuine communication. As for students' homework, students were asked to do conventional types of homework and they were never asked to express their either positive or negative emotions in relation to their learning experience. There were several indicators which showed that the two classes observed did not benefit from positive emotions. As has been pinpointed above, the classes were mostly teacher-centered and the students were jaded most of the time. This was disclosed to the observers when the learners constantly asked their teachers about when they will be dismissed. There was nearly lack of any enjoyable activities or any social bonding which could cause the learners to become engrossed by the learning process. The teachers were also wearied and fed up as they were occasionally looking at their watches which could be a sign of the lack of happiness. Moreover, the researchers did not witness an effective bond between the teachers and the learners or even amongst the learners as nobody volunteered to talk even during those short moments in which the teachers asked learners to make a contribution to the class. This could be a sign that the learners were either apprehensive or anxious that they might receive negative comments or they might be even lampooned by their teacher or classmates or they were not successful at forming positive emotional ties with their teacher and other learners to be able to talk freely. Although some students still felt that learning English takes time and they felt that they were making step by step achievements, most of the students were obsessed with negative emotions and they assumed that learning English is a herculean task. Further evidence was provided through the learners' reports about their language learning experiences. Some examples are rendered:

Learner A

*I am happy because I am learning English. My teacher is serious, but she teaches well*.

Learner B

*I am very* ***bored*** *and* ***unhappy**. I* ***don't enjoy learning**. Learning English is so hard. My teacher often asks very hard questions. I feel that I can never learn English!*

Learner C

*My teacher is not kind. She asks so many hard questions. I don't want to speak very much in the class because my scores are very low. I don't talk because my friends laugh so much on me if I want to talk*.

*Learner D*

*I am becoming better and better every day. I practice so much. Learning English is good and you should be very patient*.

Learner C

*Learning English can be hard, but now I am very happy that I can speak. Before coming to class, I couldn't speak and understand*.

Learner D

*I want my class to finish soon. There is* ***no enjoyment**. We should read all the time. My classmates are not nice. My teacher is serious*. ***I am afraid from*** *her*. ***I don't like*** *the teacher. She can be nicer……*.

Learner E

*I* ***don't have good relations with*** *my friends and teacher*. ***I think I don't learn English***. *Everything is so* ***boring*** *and so difficult. I hope we can play some games or we can laugh in the class. The book is so hard. I don't learn English very much*.

There are a lot of comments from students that show that their minds were occupied with negative emotions. Comments like “there is no enjoyment, I am afraid, everything is so boring, I am unhappy, and I do not like the teacher show sadness, fear, and anxiety” are indicators of negative emotions. Although positive emotions like optimism about learning was also extracted from the analyses of the learners' reports (e.g., they believed they were improving every day), negative emotions still outweigh positive emotions and this has its own grave consequences in terms of students' learning and their future progress. Presence of such negative emotions could prevent learners from becoming immersed in learning and gaining an acceptable proficiency. With regard to the acronym PERMA, the learners' comments were more balanced. Whereas positive comments like making day to day progress and their feelings that they could speak and the fact that they thought that learning necessitates patience are yardsticks of having a sense of achievement as well as a clear orientation and direction in learning, referring to the A (achievement) and M (meaning) parts of the acronym respectively, their reports of not establishing good relations with their classmates and teachers and their apprehension and lack of enjoyment showed absence of E (engagement) and R (relationship) parts of the PERMA.

## Discussion

Results of this study showed that teachers had higher means on IPPLL categories compared to the learners. The reason for such a finding might be that learners rarely feel the presence of positive emotions like enjoyment in their learning or if they feel such emotions, they do not assign a very significant role to such positive emotions in terms of learning. For instance, Li (2020) found out that most Chinese high school students reported moderate to high levels of trait emotional intelligence (TEI), but they also reported low to moderate levels of foreign language enjoyment (FLE). The researcher also found low to moderate correlations between students' FLE, self-perceived English achievement, and actual English achievement, meaning that the students believed that emotions like enjoyment are not likely to play a paramount role in language learning. Similarly, Jin et al. (2020) investigated whether contracting Chinese students in the foreign language could efficaciously mitigate their language anxiety. According to the contract, the students were supposed to perform voluntary speaking tasks in the class for a specified number of hours and they also guaranteed that they would express their ideas clearly and freely while having no fear from receiving any negative comments or feedback from either their classmates or teacher. After the contracting sessions on speaking, the students volunteered to answer more questions, competed with their classmates for a chance to speak, and concentrated more on what was taught. The students also showed more self-efficacy in terms of voluntarily making an oral report, practiced speaking more frequently, and displayed greater propensity to show themselves and their true capabilities. The study also revealed that the students' fear and anxiety decreased substantially after having the opportunity to prepare for the class; they got accustomed to more speaking activities and receiving positive comments and praise as they gained self-efficacy. In our study, the learners were primarily more stressed and anxious but as the treatment progressed, their feelings of anxiety and stress alleviated. Therefore, this study also provides evidence for the claim that EFL learners usually feel the presence of negative emotions excessively and these negative emotions are attenuated and weakened as they undergo PP interventions. These negative feelings usually prevent learners from thinking about positive emotions and they therefore are incognizant of effects of positive emotions in terms of a superior learning.

The lack of sufficient attention to positive emotions and lack of consistency between teachers' attitudes in the interview and their practice can be linked to a number of inherent problems in either teacher education courses or findings of research on teacher education which guide teaching and teacher education to a large extent or educational context in which learning is taking place Arbaugh et al. (2015) have found out that college of education deans believe that one of the recurrent problems of teacher preparation courses is the thin foundation of research findings which inform such programs. As Dewaele et al. (2019) have reported, after the year 2016 we have witnessed a growing interest in the role of positive emotions in both teaching and learning and positive emotions have received increasing attention in SLA through various publications as the scope of the existing research has altered and expanded to include positive emotions. Moreover, MacIntyre et al. (2019) state that both research and teaching applications of PP are proliferating rapidly and copious topics are being explored. They report that the literature on PP tends to occupy separate issues of different journals and anthologies. These findings and explanations of PP research should be inspected closely and the findings should be used as foundations upon which to design future TTC courses.

Yet another reason for overlooking positive emotions in teachers' practice might be that teachers rarely examine their own teaching practices and their beliefs about teaching and learning, a phenomenon referred to as personal epistemology. According to Pintrich (2002), personal epistemology refers to a person's cognitions about both the true nature of knowledge and learning. In other words, this kind of knowledge refers to what kind of subjective theories people tend to have about knowledge and knowing. Archer (2012) and Ryan and Bourke (2013) have proposed a framework for teachers' change. The framework emphasizes that teachers should first find a teaching issue of concern (reflection). Second, they should have an internal dialogue in which they consider structural, cultural, and personal factors including their own personal epistemology (reflexivity). They postulated that only this epistemic reflexivity would lead to decision making, also called resolved action. Relatedly, Clarke et al. (2014) found important characteristics of cooperating teachers in their study. Cooperating teachers were school teachers who assisted teacher educators and helped student teachers enter teaching profession. Another essential characteristic of cooperating teachers was that they supported reflection. That is, teacher educators must encourage teacher students to reflect upon their teaching experiences. Therefore, teachers must reflect upon their teaching practices to be able to refine and improve them. This reflection might not have taken place by the teachers in our study. In other words, the EFL teachers in the present study held agreeable and approving attitudes about the role of positive emotions in students' learning, but they were incapable of putting their beliefs into practice. The disparity between their beliefs and practice may stem from teachers' seldom examination of their prevalent views about an ideal teaching practice and the indispensable components it should embrace like emotions.

**Figure 4:**
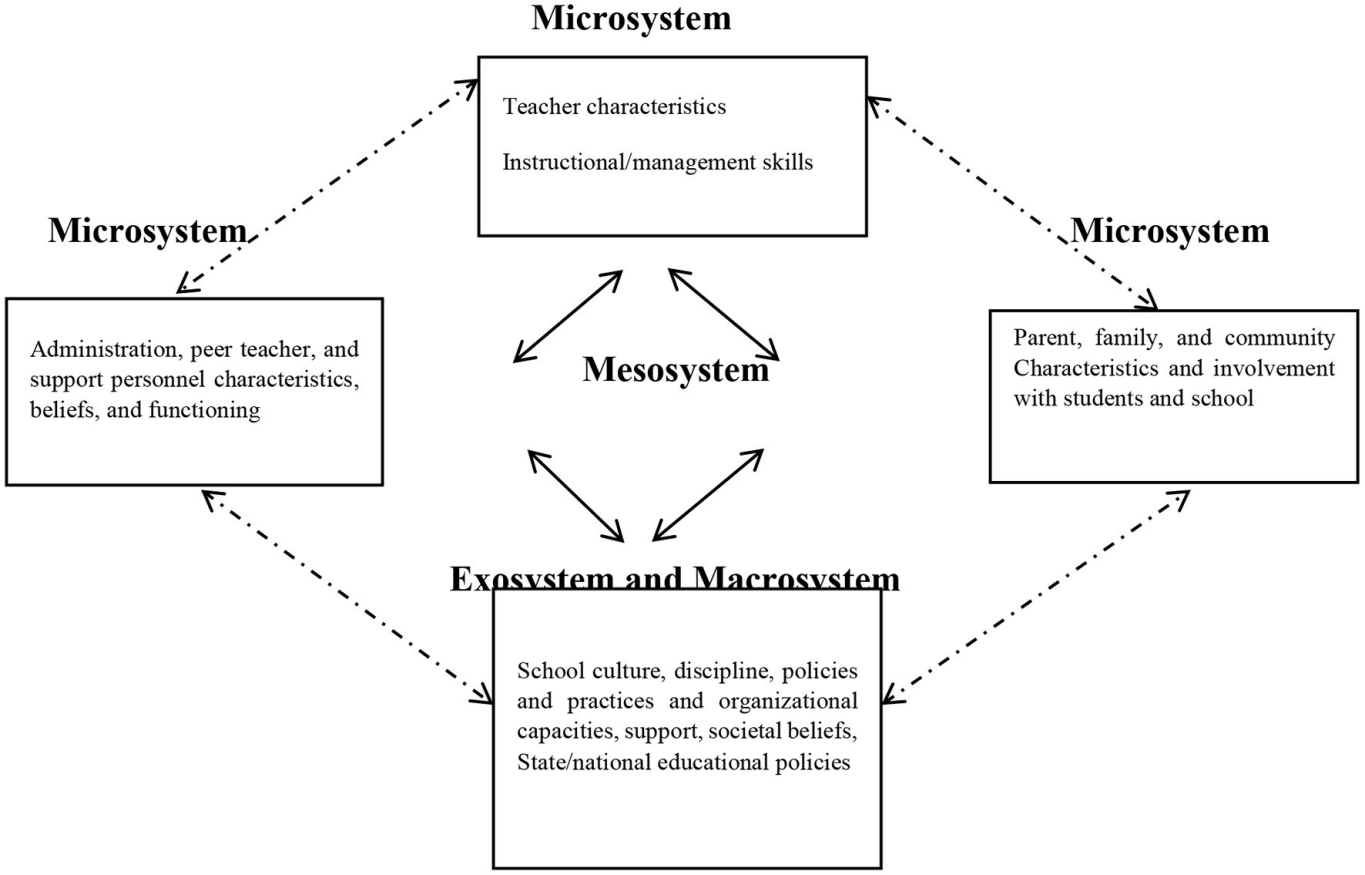
An ecological framework (Bronfenbrenner, 1979).

The other reason for why positive emotions were disregarded in the classes observed might be the context in which learning and teaching is taking place. Motoca et al. (2014) reported that it is an advisory practice to consider activities that teachers use in their classrooms through the lens of an ecological framework. When teachers are instructing students, they are affected by several factors. These factors include their colleagues, the school administration, students' parents, school board policies, neighborhood values, state-level standards, broader societal views about students, and the role of education and schooling in their development and growth. Therefore, any efforts in teacher education courses which are aimed at making changes in what teachers do should take such ecological factors in to consideration. Based on Bronfenbrenner's (1979) ecological framework, schools are considered as social ecologies which include microsystems, mesosystems, exosystems, and macrosystems (see Figure 4). Microsystem refers to all the factors which are present in immediate situation that teachers are placed in. Classroom, students' parents, and colleagues are among such factors. The mesosystems refer to linkages and connections among these various microsystems. Exosystems are events that occur outside of the teaching system that affect as well as are affected by teaching system. Policies, guidelines, and resources are all exosystems. Finally, ecosystems are the cultural contexts in which teaching and learning is taking place. Therefore, one of the reasons that positive emotions were neglected might be due to the fact that teachers are always affected by such ecological factors. Contextual expectations and standards confine what teachers are allowed to do in their classes. Resultantly, they have to select a small range of activities and classroom practices since choosing more updated activities which include attention to learners' emotional stance and dispositions can have serious repercussions of being coerced to deal with students' as well as their parents' resistance.

## Conclusion

The results of this study depicted that teachers had a higher mean in all categories in IPPLL. This finding indicates that the teachers hold a positive view toward applying PP in classrooms. Similarly, many positive emotions and PERMA elements were found in the teachers' responses to the interview questions. The results however were not congruent with the teachers' practices in the classroom. This may be due the context in which a teacher is working and all the interrelated variables which take its toll on teachers' practices. That is, teachers might have some innovative ideas at their disposal, but they cannot put them in to practice because they are petrified of parents' complaints or institutional policies or regulations since such criticisms and objections might lead to their dismissal.

Like all studies which are conducted in applied linguistics field, this study suffers from a number of limitations. First, only female teachers were interviewed and their classes were observed. Other studies can include both male and female teachers which would allow a comparison between male and female teachers' views on IPPLL. Second, due to difficulties associated with getting permission from institutes' managers, this study used convenience sampling method to collect data. Studies could be done in which IPPLL questionnaire is administered to teachers from different branches of an institute or even diverse language schools to ascertain whether teachers from different institutes judge the IPPLL items similarly. Concerning suggestions for further research, since in this study it has been discovered that teachers have favorable views toward positive emotions and therefore PP in general, studies can be carried out in which positive education is included in teachers' practices to see what effects this inclusion can have on second and foreign language acquisition. Moreover, this study demonstrated that teachers tend to agree with the IPPLL items, so future research can look into the possible relationship between the way learners answer the IPPLL items and their exam scores.

## Data Availability Statement

The raw data supporting the conclusions of this article will be made available by the authors, without undue reservation.

## Ethics Statement

The studies involving human participants were reviewed and approved by Kish Language Institute. Written informed consent to participate in this study was provided by the participants' legal guardian/next of kin.

## Author Contributions

EO conceived the research idea and wrote up the article which was read and commented on multiple times by AR and MN. EO and MN revised the article which was given final approval by AR. MN submitted the manuscript and did all the corresponding with the journal. All authors contributed to the article and approved the submitted version.

## Conflict of Interest

The authors declare that the research was conducted in the absence of any commercial or financial relationships that could be construed as a potential conflict of interest.

## Publisher's Note

All claims expressed in this article are solely those of the authors and do not necessarily represent those of their affiliated organizations, or those of the publisher, the editors and the reviewers. Any product that may be evaluated in this article, or claim that may be made by its manufacturer, is not guaranteed or endorsed by the publisher.
