# Supplementary material for: Positive Psychology and SLA Revisited: Unearthing Positive Emotions in EFL Classrooms
Source: Front Psychol. 2022 Jun 27;13:922413. doi: 10.3389/fpsyg.2022.922413 (PMC9271923; doi:10.3389/fpsyg.2022.922413)
Supplement: Supplementary file 1 [file Data_Sheet_1.docx]

**Appendix A:** The interview questions

1. In your idea what makes learners learn better?

2. What makes learners successful learners?

3. What makes a teacher a successful teacher?

4. What characteristics do all unsuccessful learners share?

5. What characteristics do all unsuccessful teachers share?

6. What characteristics do all successful learners share?

7. What characteristics do all successful teachers share?

8. What factors are deemed as important factors in teaching process?

9. What are some factors which are usually ignored in teaching and learning process?

**Appendix B:** The adapted PP Observation Checklist

| Seating arrangement that causes students to exchange more emotions and ideas with each other | Satisfactory | Promotable |
| --- | --- | --- |
| Establishing positive rapport with the learners |  |  |
| Respectful and positive environment |  |  |
| Classroom dynamics through creating positive emotions |  |  |
| Using classroom devices and realia that create positive stance within learners |  |  |
| Operative pair /Group work which involve exchange of positive emotions among learners |  |  |
| S-S Interaction that involve exchange of positive emotions |  |  |
| Monitoring students’ sense of positivity and enjoyment in classroom |  |  |
| Games with the aim of creating positive emotions |  |  |
| Activating MI through PP interventions |  |  |
| Logical pace of the lesson in a way as to increase learners’ sense of optimism about their learning |  |  |
| Wait-time as to decrease negative emotions like anxiety and stress and to make students feel happier with their learning experience |  |  |
| Reduced TTT to decrease negative emotions and to make students less bored |  |  |
| Enough STT with the aim of creating feelings like hope, optimism, and enjoyment in learners |  |  |
| Teaches the skills according to ESA (engages students’ attention with the use of positive emotions) |  |  |
| Classroom Assessment that causes students to feel more positive by reminding them of their achievements in learning |  |  |
| Correction ( that encourages students to feel more positive and optimistic about what they have learnt) |  |  |
| Personalization (paying attention to students’ emotional stance and creating positive emotions) |  |  |
| Homework (Written portfolios to encourage students to write about their emotions) |  |  |
| Progress chart (listing both learning and emotional objectives) |  |  |
| Elicitation(Brainstorming) which creates positive emotions like enjoyment in students |  |  |

**Appendix C:** The Inventory of positive psychology in language learning (IPPLL)

Dear instructors,

Read the items carefully and tick the extent to which you agree with each item.

| Strongly agree | agree | No opinion | disagree | Strongly disagree | Item |
| --- | --- | --- | --- | --- | --- |
|  |  |  |  |  | 1.Students' use of creative learning methods is effective in their learning |
|  |  |  |  |  | 2.Teacher's use of creative learning methods is effective in Students' learning |
|  |  |  |  |  | 3. Students' interest in the materials which are presented is effective in their learning. |
|  |  |  |  |  | 4. Students' interest in classmates is effective in their learning. |
|  |  |  |  |  | 5. Students' interest in their teacher is effective in their learning. |
|  |  |  |  |  | 6. Students' interest in class atmosphere is effective in their learning. |
|  |  |  |  |  | 7. Having a critical stance toward what is presented is effective in students' learning. |
|  |  |  |  |  | 8. Mastering new skills and areas is effective in students' learning |
|  |  |  |  |  | 9. Classmates' feedback are effective in students' learning. |
|  |  |  |  |  | 10. Teacher’s feedback are effective in students' learning |
|  |  |  |  |  | 11.Being able to deal with difficulties and challenges is effective in students' learning |
|  |  |  |  |  | 12.Becoming a volunteer for doing class activities is effective in students' learning |
|  |  |  |  |  | 13.Having a high level of perseverance and expanding a lot of effort is effective in students' learning |
|  |  |  |  |  | 14. Showing their true skills and capabilities in the class is effective in students' learning |
|  |  |  |  |  | 15. High attraction of classroom materials is effective in students' learning |
|  |  |  |  |  | 16. An exciting classroom is effective in students' learning |
|  |  |  |  |  | 17. Having energetic classmates is effective in students' learning. |
|  |  |  |  |  | 18.Having an energetic teacher is effective in students' learning |
|  |  |  |  |  | 19.Having a high rapport with classmates is effective in students' learning |
|  |  |  |  |  | 20. Having a high rapport with teacher is effective in students' learning |
|  |  |  |  |  | 21. Teacher’s interest in students is effective in their learning |
|  |  |  |  |  | 22. Teacher's interest in students' classmates is effective in their learning |
|  |  |  |  |  | 23.Assisting classmates is effective in students' learning |
|  |  |  |  |  | 24.Assisting teacher is effective in students' learning |
|  |  |  |  |  | 25. Students' awareness about their own feelings is effective in their learning |
|  |  |  |  |  | 26.Students' awareness about their classmates' feelings is effective in their learning |
|  |  |  |  |  | 27.Being aware of the true value of learning is effective in students' learning |
|  |  |  |  |  | 28.Activities that create motivation in me are effective in students' learning |
|  |  |  |  |  | 29.Activities that create motivation in students' classmates are effective in their learning |
|  |  |  |  |  | 30.Group activities done in classroom are effective in students' learning |
|  |  |  |  |  | 31.Getting classmates’ feedback about class activities is effective in students' learning |
|  |  |  |  |  | 32.The same behavior of teacher with all students is effective in students' learning |
|  |  |  |  |  | 33.Being a group leader is effective in students' learning |
|  |  |  |  |  | 34.Being able to accomplish group activities successfully is effective in students' learning |
|  |  |  |  |  | 35.Getting help from classmates is effective in students' learning |
|  |  |  |  |  | 36.Getting help from teacher is effective in students' learning |
|  |  |  |  |  | 37.Giving corrective feedback to their classmates is effective in students' learning |
|  |  |  |  |  | 38.Receiving corrective feedback on their mistakes from classmates is effective in students' learning |
|  |  |  |  |  | 39.Receiving corrective feedback on their mistakes from the teacher is effective in students' learning |
|  |  |  |  |  | 40. Teacher’s neglect of their mistakes is effective in students' learning |
|  |  |  |  |  | 41. Classmates’ awareness of their capabilities is effective in students' learning |
|  |  |  |  |  | 42. Teacher’s awareness of their capabilities is effective in students' learning |
|  |  |  |  |  | 43.Making conscious choices about learning methods is effective in students' learning |
|  |  |  |  |  | 44.Monitoring their own learning is effective in students' learning |
|  |  |  |  |  | 45.Monitoring their emotions is effective in students' learning |
|  |  |  |  |  | 46.Having a sense of gratitude toward classmates is effective in students' learning |
|  |  |  |  |  | 47.Having a sense of gratitude toward the teacher is effective in students' learning |
|  |  |  |  |  | 48.Being grateful about good events which have happened to them as the result of learning the language is effective in students' learning |
|  |  |  |  |  | 49.Being optimistic about learning the language fully is effective in students' learning |
|  |  |  |  |  | 50. Students' sense of humor is effective in their learning |
|  |  |  |  |  | 51.Classmates' sense of humor is effective in students' learning |
|  |  |  |  |  | 52.The teacher's sense of humor is effective in students' learning |
|  |  |  |  |  | 53.Having an awareness about goals of learning a language is effective in students' learning |
|  |  |  |  |  | 54.Having an awareness of the benefits of learning a language is effective in students' learning |

**Appendix D:** Cronbach’s Alpha Reliability Indices and Item-total Correlations for the six sub-categories of the IPPLL

| Table II  *Item-Total Statistics and Cronbach’s Alpha for Wisdom and Knowledge (Pilot Study)* | | | | |
| --- | --- | --- | --- | --- |
|  | Scale Mean if Item Deleted | Scale Variance if Item Deleted | Corrected Item-Total Correlation | Cronbach's Alpha if Item Deleted |
| PS1 | 25.31 | 47.185 | .633 | .814 |
| PS2 | 25.28 | 47.394 | .636 | .814 |
| ***PS3*** | ***25.25*** | ***54.054*** | ***.208*** | ***.853*** |
| PS4 | 25.27 | 48.140 | .568 | .820 |
| PS5 | 25.31 | 47.195 | .627 | .815 |
| PS6 | 25.28 | 47.089 | .630 | .814 |
| ***PS7*** | ***25.27*** | ***54.750*** | ***.179*** | ***.855*** |
| PS8 | 25.30 | 47.908 | .609 | .817 |
| PS9 | 25.28 | 46.906 | .628 | .814 |
| PS10 | 25.27 | 47.448 | .632 | .814 |
| Alpha | .839 |  |  |  |

| Table III  *Item-Total Statistics and Cronbach’s Alpha for Courage (Pilot Study)* | | | | |
| --- | --- | --- | --- | --- |
|  | Scale Mean if Item Deleted | Scale Variance if Item Deleted | Corrected Item-Total Correlation | Cronbach's Alpha if Item Deleted |
| PS11 | 19.61 | 33.545 | .629 | .810 |
| PS12 | 19.60 | 33.782 | .653 | .807 |
| PS13 | 19.59 | 33.841 | .621 | .811 |
| ***PS14*** | ***19.66*** | ***40.679*** | ***.135*** | ***.869*** |
| PS15 | 19.63 | 33.874 | .616 | .811 |
| PS16 | 19.58 | 32.943 | .677 | .803 |
| PS17 | 19.61 | 33.654 | .624 | .810 |
| PS18 | 19.60 | 34.401 | .609 | .813 |
| Alpha | .837 |  |  |  |

| Table IV  *Item-Total Statistics and Cronbach’s Alpha for Humanity (Pilot Study)* | | | | |
| --- | --- | --- | --- | --- |
|  | Scale Mean if Item Deleted | Scale Variance if Item Deleted | Corrected Item-Total Correlation | Cronbach's Alpha if Item Deleted |
| PS19 | 28.01 | 53.685 | .623 | .810 |
| PS20 | 28.02 | 53.822 | .626 | .810 |
| PS21 | 28.02 | 53.659 | .629 | .809 |
| ***PS22*** | ***28.09*** | ***60.887*** | ***.208*** | ***.846*** |
| PS23 | 28.01 | 53.607 | .637 | .809 |
| PS24 | 28.02 | 53.971 | .636 | .809 |
| ***PS25*** | ***28.05*** | ***61.591*** | ***.184*** | ***.846*** |
| PS26 | 28.01 | 52.825 | .647 | .807 |
| ***PS27*** | ***28.07*** | ***61.593*** | ***.183*** | ***.847*** |
| PS28 | 28.01 | 54.094 | .610 | .811 |
| PS29 | 28.07 | 53.506 | .647 | .808 |
| Alpha | . 834 |  |  |  |

| Table V  *Item-Total Statistics and Cronbach’s Alpha for Justice (Pilot Study)* | | | | |
| --- | --- | --- | --- | --- |
|  | Scale Mean if Item Deleted | Scale Variance if Item Deleted | Corrected Item-Total Correlation | Cronbach's Alpha if Item Deleted |
| PS30 | 11.20 | 14.317 | .602 | .805 |
| PS31 | 11.23 | 14.035 | .646 | .792 |
| PS32 | 11.24 | 14.167 | .626 | .798 |
| PS33 | 11.25 | 14.243 | .643 | .794 |
| PS34 | 11.24 | 14.216 | .629 | .798 |
| Alpha | .831 |  |  |  |

| Table VI  *Item-Total Statistics and Cronbach’s Alpha for Temperance (Pilot Study)* | | | | |
| --- | --- | --- | --- | --- |
|  | Scale Mean if Item Deleted | Scale Variance if Item Deleted | Corrected Item-Total Correlation | Cronbach's Alpha if Item Deleted |
| PS35 | 28.10 | 63.105 | .697 | .856 |
| PS36 | 28.10 | 62.946 | .696 | .856 |
| PS37 | 28.11 | 63.180 | .696 | .857 |
| ***PS38*** | ***28.10*** | ***73.125*** | ***.159*** | ***.892*** |
| PS39 | 28.12 | 63.771 | .669 | .858 |
| ***PS40*** | ***28.12*** | ***73.800*** | ***.123*** | ***.894*** |
| PS41 | 28.06 | 63.080 | .676 | .858 |
| PS42 | 28.10 | 63.781 | .678 | .858 |
| PS43 | 28.09 | 63.953 | .674 | .858 |
| PS44 | 28.09 | 62.974 | .721 | .855 |
| PS45 | 28.11 | 63.774 | .691 | .857 |
| Alpha | . 875 |  |  |  |

| Table VII  *Item-Total Statistics and Cronbach’s Alpha for Transcendence (Pilot Study)* | | | | |
| --- | --- | --- | --- | --- |
|  | Scale Mean if Item Deleted | Scale Variance if Item Deleted | Corrected Item-Total Correlation | Cronbach's Alpha if Item Deleted |
| PS46 | 22.59 | 43.045 | .599 | .818 |
| PS47 | 22.54 | 43.258 | .560 | .822 |
| PS48 | 22.56 | 42.631 | .623 | .815 |
| PS49 | 22.55 | 42.872 | .604 | .817 |
| PS50 | 22.59 | 42.660 | .594 | .818 |
| PS51 | 22.58 | 42.055 | .657 | .811 |
| PS52 | 22.59 | 42.300 | .630 | .814 |
| ***PS53*** | ***22.57*** | ***50.280*** | ***.123*** | ***.866*** |
| PS54 | 22.55 | 42.174 | .601 | .817 |
| Alpha | .840 |  |  |  |
